# Medicinal Insights Into FLT3 Inhibitors as Anticancer Agents: Current Status and Future Direction

**DOI:** 10.1002/ardp.70302

**Published:** 2026-07-08

**Authors:** Ankush Kumar, Pallvi Kumari, Keshav Raj Paudel, Rajwinder Kaur, Rohit Bhatia

**Affiliations:** ^1^ Chitkara College of Pharmacy Chitkara University Rajpura Punjab India; ^2^ School of Pharmaceutical Sciences CT University Ludhiana Punjab India; ^3^ NICM Health Research Institute and School of Science Western Sydney University Westmead New South Wales Australia

**Keywords:** AML, cancer, FLT3 inhibitors, SAR, tyrosine kinase

## Abstract

FMS‐like tyrosine kinase 3 (FLT3) is a receptor tyrosine kinase (RTK) important for hematopoietic stem cell proliferation, differentiation, and survival. Ligand‐induced dimerization activates the downstream pathways such as RAS/MAPK, PI3K/AKT, and STAT5. Mutations such as internal tandem duplications (ITD) and tyrosine kinase domain (TKD) cause constitutive activation, poor prognosis, and driving leukemogenesis in acute myeloid leukemia (AML). Over the past 5 years, FLT3‐targeted therapy has advanced significantly. Second‐generation FLT3 inhibitors such as gilteritinib and quizartinib displayed improved selectivity and durable efficacy. Quizartinib was approved in 2023 for FLT3‐ITD–positive AML alongside intensive chemotherapy. Clinical trials have explored some compounds such as crenolanib, momelotinib, and various novel molecules in combination regimens, which are enhancing remission rates. This review comprehensively compiles diverse chemical scaffolds investigated as FLT3 inhibitors such as pyrimidine, benzimidazole, imidazole, indole, isoxazole, quinazoline, and so on. Structure–activity relationship (SAR) analyses and molecular docking studies are briefly discussed along with highlighting potency against FLT3‐ITD and resistant FLT3‐TKD mutants. Despite therapeutic gains, resistance through secondary mutations or compensatory pathway activation remains a challenge. Future directions should focus on structure‐guided design, rational combination therapies, and expanding applications to other FLT3‐altered malignancies. This review integrates updated FLT3 biology, clinical outcomes, medicinal chemistry, and computational insights to support personalized FLT3‐targeted treatment strategies.

## Graphical abstract

Structure–activity relationship of FMS‐like tyrosine kinase 3 (FLT3) as potent anti‐cancer agents supported by biological evaluation, in silico and clinical trial studies.

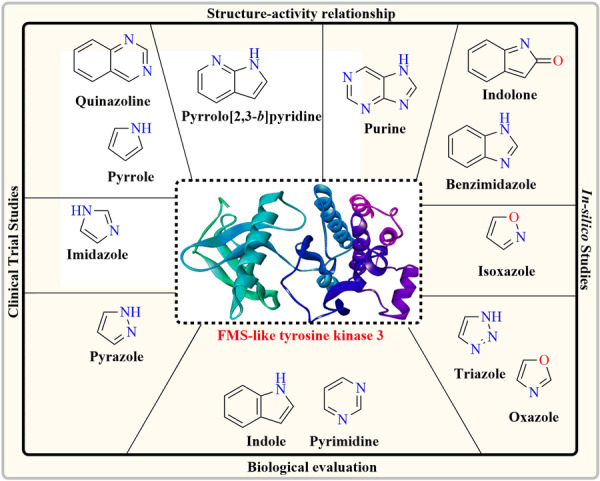

**Abbreviations**
AML: acute myeloid leukemia
FLT3: FMS‐like tyrosine kinase 3
FL: FLT3 ligand
ITD: internal tandem duplications
SAR: Structure‐activity relationship
TKD: tyrosine kinase domain

## 1 Introduction

Acute myeloid leukemia (AML) is a biologically complex and clinically aggressive malignancy of the hematopoietic system characterized by clonal proliferation of undifferentiated myeloid precursors [1, 2]. It is one of the most common forms of leukemia in adults and poses a significant global health challenge. Based on the latest epidemiological data, AML accounts for approximately 1.1% of all new cancer cases worldwide, with an estimated 475,000 new diagnoses and over 311,000 deaths annually [3]. The incidence of AML increases markedly with a median age at diagnosis of 68–70 years, and the burden is projected to rise further with global population aging [4].

AML incidence increases with age, and the prognosis remains poor in older adults, with a 5‐year survival rate below 30%, largely due to frequent relapse, chemoresistance, and underlying genetic heterogeneity [5]. Disease onset is often rapid, and untreated AML can lead to death within weeks to months due to bone marrow failure, severe infections, or bleeding complications [6]. The disease is profound biological diversity, reflected in its varied cytogenetic and molecular abnormalities. It has significant implications for prognosis and therapy selection. Traditional chemotherapy agents including cytarabine and anthracycline‐based induction therapies have shown limited long‐term success in many patient subgroups, emphasizing the urgent need for more precise and targeted therapeutic strategies [7]. Although these regimens can achieve high initial remission rates, but the majority of patients particularly those with adverse‐risk genetic profiles experience relapse. These limitations have boosted the shift toward precision medicine approaches designed to exploit specific molecular vulnerabilities within AML cells.

Recent advancements in molecular profiling of AML have led to the identification of several major genetic mutations and cytogenetic abnormalities that contribute to disease initiation, progression, and therapeutic resistance [8]. These include mutations in NPM1, FLT3, CEBPA, IDH1/2, DNMT3A, and TP53, as well as recurrent chromosomal translocations [9, 10]. Among these, FLT3 mutations represent one of the most prevalent and clinically significant aberrations, occurring in approximately 30%–35% of AML cases [11]. FLT3 encodes the FMS‐like tyrosine kinase 3 receptor. It is a critical regulator of normal hematopoietic stem cell proliferation, differentiation, and survival [12, 13]. Mutations, most commonly FLT3‐internal tandem duplications (FLT3‐ITDs) and FLT3‐tyrosine kinase domain (FLT3‐TKD) point mutations, result in constitutive activation of the receptor, driving uncontrolled leukemic cell growth and survival [14]. These mutations serve not only as key drivers of leukemogenesis but also as prognostic biomarkers, being associated with increased relapse risk and poor survival outcomes [15, 16]. The adverse impact is especially pronounced in FLT3‐ITD mutations with high allelic ratios. FLT3 mutations have become attractive therapeutic targets because of their frequent and prognostic importance. The development of selective FLT3 inhibitors (both as monotherapy and in combination with conventional chemotherapy) have demonstrated promising clinical outcomes and represents a pivotal advance in AML treatment.

The present review summarizes recent advancements in development, SAR studies, in‐silico investigations, and biological evaluations of FLT3‐targeted compounds to provide insights into the rational design of potent and selective inhibitors for AML therapy.

## 2 Structural Representation and Functional Biology of FLT3

**Figure 1:**
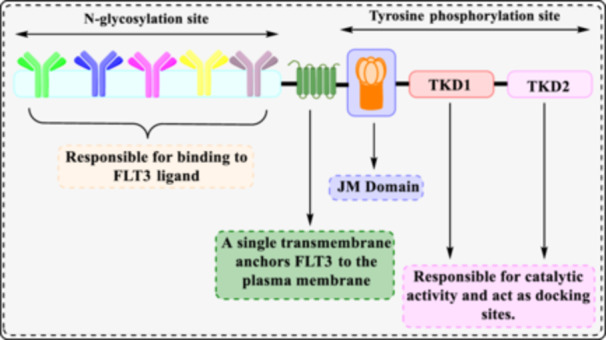
Structural representation of FLT3 protein.

FLT3 is composed of an extracellular domain, transmembrane segment, juxtamembrane (JM) region, and a split intracellular tyrosine kinase domain [17] (FLT3‐TKD1 and FLT3‐TKD2, as displayed in Figure 1). The extracellular portion contains five immunoglobulin‐like domains, which specifically mediate binding to the FLT3 (FL) ligand [18]. Ligand binding induces receptor dimerization, a key step for the activation of its kinase function. The transmembrane domain serves as a single hydrophobic anchor, securing the receptor within the plasma membrane, while the JM region acts as a regulatory segment that keeps the receptor in an inactive conformation under basal conditions. Upon ligand‐induced activation, conformational changes relieve JM‐mediated autoinhibition, enabling the intracellular tyrosine kinase domain to undergo trans‐autophosphorylation at specific residues such as Y589, Y591, and Y842 [19, 20]. These phosphorylation events initiate a cascade of downstream signaling processes.

Biologically, FLT3 is predominantly expressed in hematopoietic stem and progenitor cells and is essential for their proliferation, differentiation, and survival. Its activation stimulates multiple signaling networks, including the PI3K/AKT pathway for cell survival, the RAS/MAPK pathway for proliferation, and the STAT5 pathway for transcriptional regulation of growth‐related genes [21, 22]. These mutations cause ligand‐independent constitutive activation of FLT3, leading to persistent downstream signaling, uncontrolled myeloid proliferation, impaired differentiation and enhanced resistance to apoptosis. These mutations lead to constitutive activation of the receptor, independent of ligand binding, promoting uncontrolled cell proliferation and poor clinical prognosis.

## 3 Therapeutic Targeting of FLT3

FLT3 plays a pivotal role in regulating haematopoiesis by controlling cell survival, proliferation, and differentiation. Under normal physiological conditions, FLT3 activation is strictly ligand‐dependent. The FLT3 ligand (FL), a cytokine produced primarily by bone marrow stromal cells, binds to the extracellular domain of the receptor, inducing receptor dimerization [13]. This conformational change triggers autophosphorylation of specific tyrosine residues within the JM and FLT3‐TKDs, thereby activating the receptor. Following activation, FLT3 recruits and stimulates multiple downstream signaling cascades including the RAS/MAPK, the PI3K/AKT, and the JAK/STAT pathway [23, 24]. These pathways collectively promote cellular outcomes such as proliferation, metabolic regulation, anti‐apoptotic signaling, and lineage commitment during normal haematopoiesis. In the absence of FL, the receptor remains monomeric and inactive, resulting in no significant downstream signaling [18].

**Figure 2:**
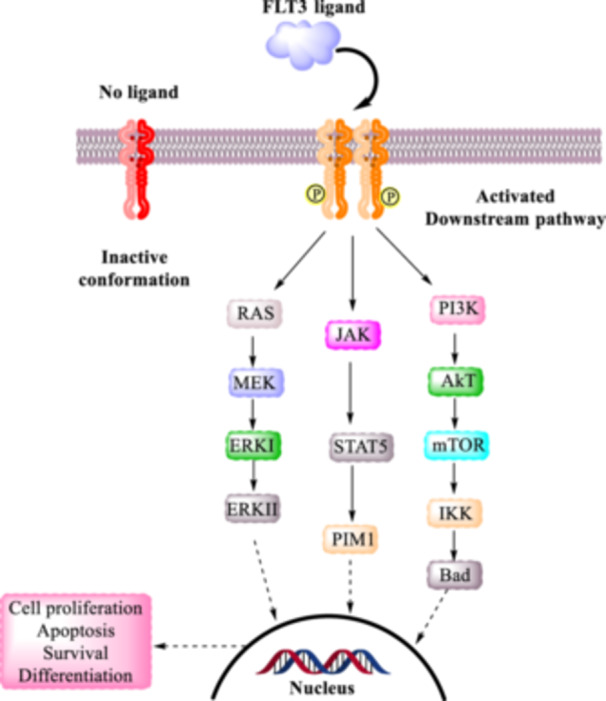
FLT3‐driven signaling pathways in cancer.

However, in pathological states such as AML, somatic mutations within the FLT3 gene, most frequently FLT3‐ITDs in the JM region or point mutations in the FLT3‐TKD lead to ligand‐independent constitutive activation [25]. These gain‐of‐function mutations mimic the active conformation of ligand‐bound FLT3, resulting in persistent autophosphorylation and continuous stimulation of downstream signaling pathways, even in the absence of ligand. This aberrant signaling disrupts the normal balance of cell proliferation and apoptosis, leading to uncontrolled clonal expansion of leukemic blasts and impaired differentiation [26, 27]. Moreover, constitutive FLT3 signaling can also promote genomic instability, therapy resistance, and disease progression [28]. Constitutive FLT3 signaling not only promotes uncontrolled clonal expansion of leukemic blasts but also contributes to genomic instability, therapy resistance, and disease progression. Figure 2 illustrates the canonical signaling pathways regulated by FLT3 in both its physiological and mutated states. Given its central role in AML pathogenesis, FLT3 has emerged as a highly attractive therapeutic target.

Small‐molecule FLT3 inhibitors have been developed to block kinase activity, thereby interrupting aberrant signaling and inducing leukemic cell death [29]. FLT3 inhibitors are broadly classified into Type I and Type II agents, based on their binding conformation within the kinase domain [19, 30]. This distinction has critical therapeutic and resistance implications. To date, three FLT3 inhibitors (summarized in Table 1) have received US FDA approval [34]. Type I inhibitors bind to the active (DFG‐in) conformation of the FLT3 kinase domain, although some may also accommodate inactive states. Because of this flexible binding mode, they maintain inhibitory activity against both FLT3‐ITD and most FLT3‐TKD mutations, including the clinically relevant D835 activation loop mutation [35]. Clinically approved examples include midostaurin and gilteritinib, while crenolanib remains under active clinical investigation.

**Table 1:** FDA‐approved FLT3 inhibitors for the treatment of AML.

S. no.	Drug name	Brand name	Chemical structure	Approval year	Indication	FLT3 mutation targeted	Type	Mechanism	Reference
1	Midostaurin	Rydapt	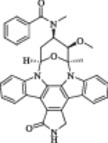	2017	Newly diagnosed AML (FLT3^+^)^+^ chemo	FLT3‐ITD, FLT3‐TKD	Type I inhibitor	Multi‐kinase (FLT3, KIT, PDGFR)	[31]
2	Gilteritinib	Xospata	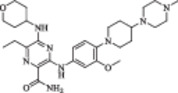	2018	R/R AML with FLT3 mutation	FLT3‐ITD, FLT3‐TKD	Type I selective	Potent FLT3/AXL dual inhibitor	[32]
3	Quizartinib	Vanflyta	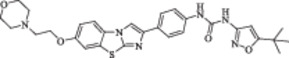	2023	FLT3‐ITD AML (combo)	FLT3‐ITD	Type II selective	Selective FLT3‐ITD inhibitor	[33]

Midostaurin is a first‐generation semi‐synthetic multikinase inhibitor (Rydapt) that have received US FDA approval in April 2017 for newly diagnosed AML with FLT3‐ITD or TKD mutations, administered alongside standard induction (cytarabine + daunorubicin) and consolidation chemotherapy, with optional maintenance [31, 36]. Preclinical studies show it potently inhibits FLT3 (low‑nanomolar IC_50_), blocks autophosphorylation, and suppresses STAT5, AKT, and ERK signaling, leading to apoptosis in FLT3‑mutant AML cell lines and reduced tumor burden in xenograft models [37, 38, 39]. In the pivotal phase III RATIFY trial (CALGB 10603), adding midostaurin to chemotherapy significantly improved event‑free survival (EFS: 8.2 vs. 3.0 months, HR: 0.78) and overall survival (median OS: 74.7 vs. 25.6 months) [39]. Benefits were observed across FLT3‑ITD and FLT3‐TKD subgroups and were more found in males. Patients undergoing allogeneic HSCT in first remission had 56% 10‑year OS with midostaurin versus 36% without transplant [37, 40]. Common adverse effects include gastrointestinal symptoms, rash, transient cytopenias, and rare cardiac toxicities such as perimyocarditis have been reported [41].

Gilteritinib (Xospata) is a selective second‐generation FLT3 inhibitor with potent activity against both FLT3‐ITD and FLT3‐TKD mutations and was approved by the US FDA in November 2018 for relapsed or refractory (R/R) AML as monotherapy [32, 42, 43]. Developed by Astellas Pharma, it is a pyrazine‐carboxamide derivative that binds the ATP‐binding pocket of FLT3 and interacts with the gatekeeper F691 residue, stabilizing the inactive conformation [44]. In the phase III ADMIRAL trial, gilteritinib significantly improved median OS compared with salvage chemotherapy (9.3 vs. 5.6 months; HR = 0.64) in FLT3‐mutated R/R AML, with higher complete remission/complete remission with partial hematologic recovery (CR/CRh) rates (34% vs. 15%) [42]. Common toxicities include cytopenias, transaminase elevations, and differentiation syndrome [42].

Type II inhibitors bind exclusively to the inactive (DFG‐out) conformation of FLT3. While these agents demonstrate strong and selective activity against FLT3‐ITD mutations, their activity is substantially compromised in the presence of FLT3‐TKD mutations, particularly the D835 substitution [45]. This limitation accounts for frequent resistance observed during treatment.

Quizartinib (Vanflyta), a highly selective FLT3‐ITD inhibitor with minimal FLT3‐TKD activity, received FDA approval in July 2023 for newly diagnosed FLT3‐ITD–positive AML in combination with intensive chemotherapy [33, 46]. By occupying the ATP‐binding cleft of FLT3, quizartinib effectively blocks kinase activation and downstream oncogenic signaling [47]. In the phase III QuANTUM‐First trial, adding quizartinib to chemotherapy improved median OS (31.9 vs. 15.1 months; HR = 0.78) without compromising remission rates, with manageable toxicities such as QT prolongation and myelosuppression [48].

Collectively, these inhibitors midostaurin, gilteritinib, and quizartinib act by blocking ATP binding and suppressing kinase activity of mutant FLT3, thereby interrupting downstream pro‐leukemic pathways (RAS/MAPK, PI3K/AKT, and STAT5) and improving clinical outcomes in defined patient subsets.

The FLT3‐F691L gatekeeper mutation further decreases the effectiveness of many inhibitors by altering the ATP‐binding site, leading to resistance to several approved drugs. However, recent studies have reported new compounds that can still inhibit FLT3 carrying the F691L mutation. Overall, these limitations contribute to the frequent development of resistance during treatment.

## 4 Clinical Trial Status of Drugs Targeting FLT3

**Figure 3:**
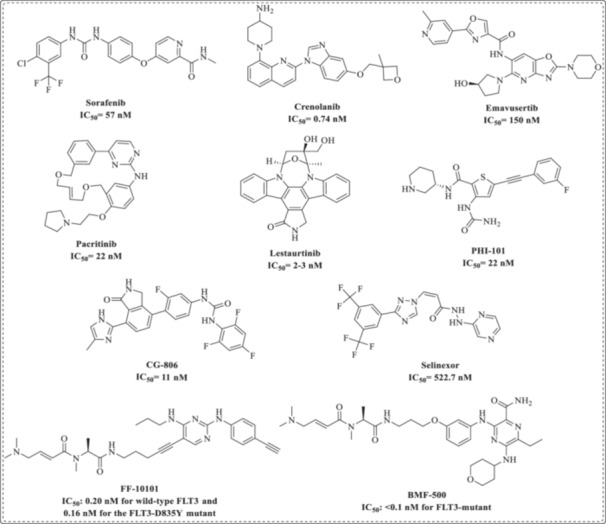
FTL3 inhibitors currently under clinical trials.

Over the past decade, several FLT3 inhibitors have entered clinical development with varying degrees of selectivity and efficacy. First‐generation inhibitors such as midostaurin and sorafenib have demonstrated moderate clinical benefits and received approval for use in FLT3‐mutated AML, particularly in combination with standard chemotherapy. Subsequently, second‐generation inhibitors such as gilteritinib, quizartinib, and crenolanib have shown improved potency and specificity against FLT3 mutations. Gilteritinib has been approved for relapsed or refractory FLT3‐mutated AML, while quizartinib has recently gained regulatory approval in selected regions following successful Phase III trials. Numerous ongoing clinical trials are now evaluating the efficacy of these agents in combination therapies in newly diagnosed patients (displayed in Table 2). NCT06303193 is a Phase I/II trial sponsored by the National Cancer Institute (NCI) to evaluate pacritinib, an oral kinase inhibitor targeting FLT3 in adolescents (12–17 years) and adults (≥ 18 years) with myelodysplastic syndromes (MDS) or MDS/myeloproliferative neoplasms (MDS/MPN). Although not an AML trial, FLT3 mutations and overexpression are implicated in progression from MDS to AML. Pacritinib inhibits FLT3 kinase activity with an IC_50_ of 22 nM in vitro [49]. FF‐10101 is a first‐in‐class covalent inhibitor which is designed to overcome resistance associated with currently available FLT3‐targeted therapies. It works by forming a covalent bond with the FLT3 kinase [50]. It leads to sustained inhibition, which helps in overcoming resistance mutations such as the F691L gatekeeper mutation. A first‐in‐class, open‐label Phase 1 trial (NCT03194685) investigated the safety and effectiveness of the irreversible FLT3 inhibitor FF‐10101. This is a first dose‐escalation study where patients received oral FF‐10101 at different dose levels [51, 52]. BMF‐500 is a third‐generation covalent FLT3 inhibitor developed by Biomea Fusion. It is designed to target both primary and secondary FLT3 mutations, including the F691L mutation [53]. It forms a permanent covalent bond with the FLT3 target and inhibits FLT3 activity, and also maintains cytotoxicity even in the presence of the F691L mutation. Preclinical studies showed that BMF‐500 has high picomolar affinity for FLT3. A phase 1 clinical study, COVALENT‐103 (NCT05918692), is being carried out in adults with acute leukemia to determine the safety and effectiveness of BMF‐500. This is a dose‐escalation and dose‐expansion study in adult patients with AML [54, 55, 56]. Preclinical and early clinical data indicate that it suppresses FLT3‐ITD and FLT3‐TKD mutants and shows safety and initial anti‐leukemic activity when combined with chemotherapy. Figure 3 displays the chemical structures and IC_50_ values of FLT3 inhibitors in clinical trials.

**Table 2:** FLT3 inhibitors in clinical trial.

S. no.	Clinical trial number	Status	Intervention	Sponsor	Phase	Enrollments	Start date	Completion date
1.	NCT06303193	Not yet recruiting	Pacritinib	NCI	Phase II	160	07‐2025	01‐2035
2.	NCT05947344	Recruiting	STI‐8591	Zhejiang ACEA Pharmaceutical Co. Ltd.	Phase I	84	12‐2023	12‐2025
3.	NCT05918692	Recruiting	BMF‐500	Biomea Fusion Inc.	Phase I	84	07‐2023	12‐2025
4.	NCT05886049	Recruiting	Revumenib	NCI	Phase I	28	06‐2024	12‐2017
5.	NCT05756777	Recruiting	Gilteritinib|	Memorial Sloan Kettering Cancer Center	Phase I	36	06‐2023	02‐2026
6.	NCT05028751	Terminated	Gilteritinib	Kronos Bio	Phase I/II	24	08‐2022	04‐2024
7.	NCT04842370	Unknown	PHI‐101	Seoul National University Hospital	Phase I	42	06‐2020	05‐2022
8.	NCT04477291	Terminated	CG‐806	Aptose Biosciences Inc.	Phase I	45	10‐2020	04‐2024
9.	NCT04278768	Recruiting	Emavusertib	Curis Inc.	Phase I/II	366	07‐2020	04‐2026
10.	NCT03922100	Terminated	NMS‐03592088	Nerviano Medical Sciences	Phase I/II	63	04‐2019	08‐2024
11.	NCT02997202	Completed	Gilteritinib	Astellas Pharma Global Development Inc.	Phase III	356	08‐2017	05‐2023
12.	NCT02675478	Completed	AC220	Daiichi Sankyo Co. Ltd.	Phase I	17	02‐2016	11‐2018
13.	NCT02530476	Completed	Selinexor	M.D. Anderson Cancer Center	Phase I/II	17	12‐2015	04‐2019
14.	NCT01468467	Completed	AC220	Daiichi Sankyo	Phase I	13	04‐2012	03‐2015
15.	NCT01390337	Completed	AC220|	Daiichi Sankyo	Phase I	19	10‐2011	02‐2015
16.	NCT00989261	Completed	AC220	Daiichi Sankyo	Phase I	333	11‐2009	12‐2014
17.	NCT00462761	Completed	AC220	Daiichi Sankyo	Phase I	76	01‐2007	12‐2009
18.	NCT00373373	Completed	Sorafenib|	University Hospital Muenster	Phase II	200	09‐2006	07‐2009

Multiple completed clinical trials have evaluated FLT3 inhibitors across various AML context. The RATIFY trial (NCT01468467) demonstrated that adding midostaurin to standard chemotherapy significantly improved overall survival in newly diagnosed FLT3‐mutated AML [57]. In NCT02997202, gilteritinib used as post‐transplant maintenance improved relapse‐free survival in FLT3‐ITD AML patients.

Quizartinib, evaluated across several trials (NCT00373373, NCT00462761, NCT00989261, NCT01390337, NCT02675478), showed strong single‐agent and combination efficacy, with high composite complete remission rates and manageable safety profiles, especially in relapsed/refractory or newly diagnosed AML. The Phase I study (NCT01390337) combining quizartinib with 7 + 3 chemotherapy yielded an ORR of over 80% [58], while NCT00989261 and NCT00462761 established quizartinib's tolerability and dosing [59, 60]. In NCT02675478, Japanese patients treated with quizartinib achieved a 37.5% CRC rate. Finally, the combination of sorafenib and selinexor (NCT02530476) produced clinical responses in pretreated FLT3‐mutated AML. This highlights the potential of FLT3‐targeted combinations. Collectively, these trials have shaped the therapeutic landscape for FLT3‐mutated AML supporting the use of targeted agents in both frontline and relapsed contexts.

## 5 Recent Progress in the Medicinal Chemistry of FLT3 Inhibitors

Development of potent and selective FLT3 inhibitors has driven medicinal chemists to explore a wide range of heterocyclic scaffolds, each offering distinct binding modes, physicochemical profiles and resistance‐overcoming potential. Scaffold‐based classification not only facilitates an understanding of SAR but also provides a logical framework for tracing the evolution of FLT3‐targeted drug design. Early inhibitors were often derived from multi‐kinase chemotypes such as quinazolines and indolinones, whereas later generations have developed more selective heterocycles including pyrimidines, triazines, and indazole engineered to improve kinase selectivity, address secondary mutations (e.g., D835Y, F691L) and optimize PK properties. In the following subsections, recent advances have been discussed according to their core scaffold type, highlighting representative examples, SAR trends, and outcome of the particular study.

### 5.1 Pyrimidine as FLT3 Inhibitors

**Figure 4:**
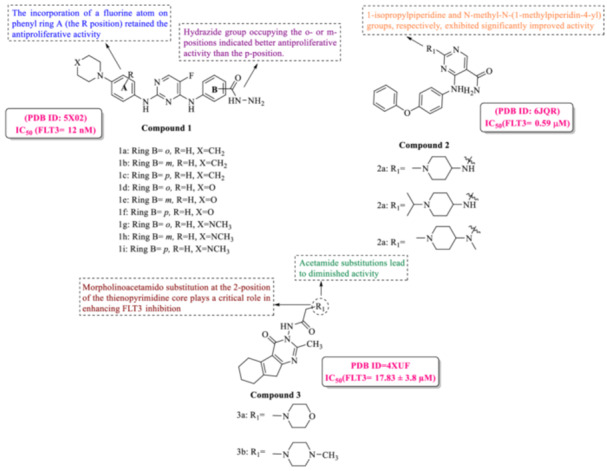
Chemical structure and SAR of Pyrimidines as FLT3 inhibitor.

The study reports the design and evaluation of hydrazido‐arylaminopyrimidines as dual BTK/FLT3 inhibitors, with **1 g** (displayed in Figure 4) emerging as the most potent lead. It inhibited BTK (IC_50_ = 47 nM) and FLT3 (IC_50_ = 12 nM) and showed strong antiproliferative effects in cancer cell lines, including Jeko‐1 (IC_50_ = 17 nM) and MV‐4‐11 (IC_50_ = 3 nM), while in vivo it achieved tumor growth inhibition of 79.8% (Jeko‐1) and 94.8% (MV‐4‐11) with good tolerability. Docking studies using BTK (PDB: 5P9L) and FLT3 (PDB: 5×02) revealed that the aminopyrimidine core of **1g** formed hinge hydrogen bonds with Glu475, Met477 (BTK), and hydrazide interactions with Ser538, Asp539, while in FLT3 it established hydrogen bonds with Leu616, Asp698, Cys828, and H–π interactions with Val624, Leu616, Gly697. The N‐methylpiperazinyl group extended toward the solvent or back pocket, enhancing binding [61].

Wei Liu and colleagues reported 3‐amide‐pyrimidine‐based derivatives by using scaffold hopping and structure simplification of G‐749. Compound **2b** (displayed in Figure 4) was the most effective of the synthesized compounds, demonstrating strong kinase inhibition against FLT3‐ITD (IC_50_ = 6.5 nM) and FLT3‐D835Y (IC_50_ = 10.3 nM). Compound **2b** also displayed strong antiproliferative activity against FLT3‐ITD‐positive MV4‐11 cells (IC_50_ = 0.59 μM). By downregulating p‐FLT3, p‐STAT5, p‐ERK, and p‐AKT signaling, **2b** mechanistically altered mitochondrial membrane potential, lowered reactive oxygen species (ROS), caused apoptosis, and stopped the cell cycle at the G0/G1 phase. Molecular docking studies with the FLT3 crystal structure (PDB ID: 6JQR) showed that **2b** made hydrophobic contacts with Phe830 and Val624, two hydrogen bonds with Glu692 and Cys694, and other interactions with Tyr696 and Leu616 [62].

A series of thieno[2,3‐d]pyrimidine acetamide derivatives (**3a)** were synthesized and evaluated for their ability to inhibit FLT3 kinase. With an IC_50_ value of 17.83 ± 3.8 µM, compound **3a** was the most active member of the series, according to the biological assay. The crystal structure of FLT3 (PDB ID: 4XUF) was used in molecular docking studies to explain these findings. Compound **3a**, with a docking score of −6.831 kcal/mol, showed enhanced enzymatic activity that was probably influenced by the strong hydrogen bonds it formed with Leu616 and Cys694 as well as extra interactions with Asp698. These results demonstrate that **3a** (displayed in Figure 4) was the most effective inhibitor according to the biological experiments, highlighting the significance of combining computational and experimental methods [63].

### 5.2 Benzimidazole as FLT3 Inhibitors

**Figure 5:**
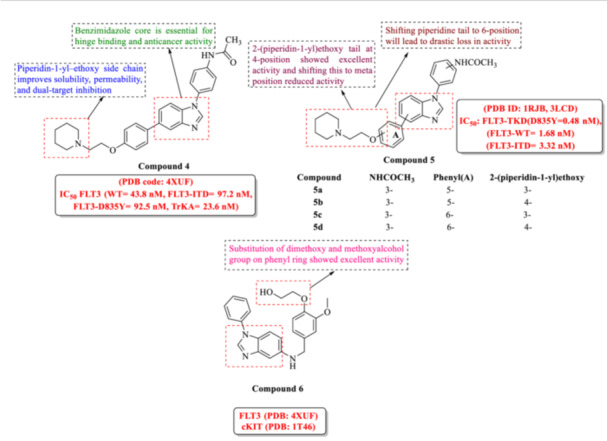
SAR of benzimidazole derivatives.

Benzimidazole derivative **4** (displayed in Figure 5) was synthesized and tested as a dual FLT3/TrKA inhibitor that targets AML. The multi‐target action of the compound was confirmed by enzyme assays, which indicated inhibition with an IC_50_ of FLT3 (WT 43.8 nM, FLT3‐ITD 97.2 nM, FLT3‐D835Y 92.5 nM) and TrKA (23.6 nM). In vitro results displayed broader action against the NTRK1 fusion‐positive KM12 colon cancer cells (358 nM) by specifically suppressing FLT3‐driven AML cell lines with IC_50_ of 38.8 ± 10.7 nM (MV4‐11) and 54.9 ± 4.1 nM (MOLM‐13). These results were confirmed by docking studies in the FLT3 inactive conformation (PDB ID: 4XUF**)**. Docking results displayed that the solvent tail connected with Cys695, the acetamido group made interaction with Asp829, and the benzimidazole core formed a hinge hydrogen bond with Cys694 [64].

A novel class of benzimidazole derivatives were synthesized and tested for anticancer properties against FLT3. Compound **5b** (Figure 5) was the most effective inhibitor in this series, exhibiting sub‐nanomolar activity against the resistant mutant FLT3‐TKD (D835Y) with an IC_50_ of 0.48 nM and high activity against FLT3‐WT (IC_50_ = 1.68 nM) and FLT3‐ITD (IC_50_ = 3.32 nM). Using IC_50_ values of 16.1 nM in MOLM‐14 and 10.5 nM in MV4‐11 AML cell lines, **22b** showed selective antiproliferative effects in cellular experiments. It also maintained notable activity against resistant sublines, MOLM‐14‐D835Y (26.5 nM) and MOLM‐14‐F691L (160.3 nM). **5b** caused G0/G1 cell‐cycle arrest and apoptosis by blocking FLT3 phosphorylation and downstream signaling (ERK, STAT5, and S6). FLT3‐TKD (D835Y) has an approximately 80‐fold higher selectivity than KIT, according to selectivity profiling, which suggests a lower probability of myelosuppression. The increased potency and stability of **5b** can be explained by its ability to make stable contacts with Cys694, Lys644, and Phe830 while fitting into the ATP‐binding site without breaking the Lys644–Glu661 salt bridge, as demonstrated by docking into a homology model of FLT3 (PDB ID: 1RJB and 3LCD) [65].

Benzoimidazole compounds were synthesized and tested for its ability to inhibit the growth of selective FLT3. The authors evaluated and contrasted the cell‐based cytotoxicity with that of the isogenic leukemic cell line and the normal cell line (BaF3). Benzoimidazole scaffold‐based compound **6** (displayed in Figure 5) was shown to be the most selective and potent. Strong binding inside the kinase active site was shown by the compound **6** through docking investigation. Compound **6** filled the hydrophobic pocket against FLT3 (PDB: 4XUF**)** and interacted with important residues Met664 and Met665 to stabilize its location. Conversely, interactions involving Val643 and Leu644 were seen with cKIT (PDB: 1T46), indicating decreased selectivity. These findings demonstrate that compound **6** uses crucial hydrophobic interactions to preferentially stabilize FLT3 binding [66].

### 5.3 Pyrrolo[2,3‐*b*]Pyridine as FLT3 Inhibitors

**Figure 6:**
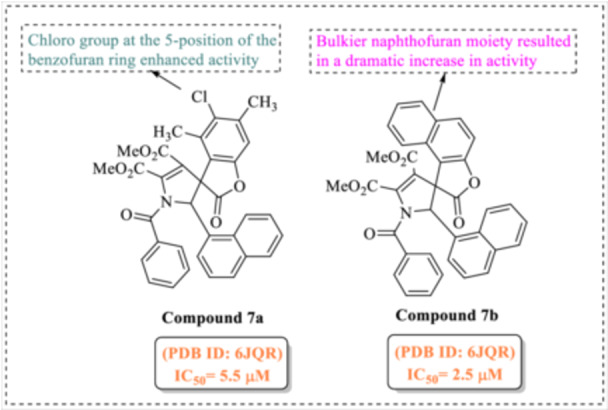
SAR of spiro[benzofuran‐3,3′‐pyrroles] derivatives.

A number of spiro[benzofuran‐3,3′‐pyrroles] derivatives (**7a–b**) were synthesized and their anticancer potential against FLT3 kinase was evaluated. The most active of them was **7b**, with an IC_50_ of 2.5 μM, then **7a** with an IC_50_ value of 5.5 μM. Both **7a** and **7b** had notable activity. Using the crystal structure of FLT3 in association with gilteritinib (PDB ID: 6JQR), molecular docking was carried out. In contrast to gilteritinib (–8.81 kcal/mol), the docking results showed binding energies ranging from −6.54 kcal/mol (**7a)** to –7.46 kcal/mol (**7b)**. Increased activity of compound **7b** was probably caused by the important hydrogen bonds it made with Asn701 and the substantial hydrophobic interactions it showed with Leu616, Val624, Gly697, and Leu818. Figure 6 shows the SAR of spiro[benzofuran‐3,3′‐pyrroles] derivatives [67].

### 5.4 Imidazole Based Derivatives

**Figure 7:**
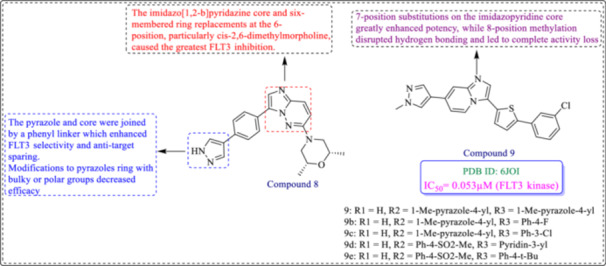
Structure and SAR of imidazole derivatives as FLT3 inhibitors.

Compound **8** (displayed in Figure 7) was discovered by Desmond Akwata et al. to be a highly selective FLT3 inhibitor. It demonstrated strong efficacy against FLT3 while exhibiting low inhibition of important anti‐target kinases, including VEGFR2 (KDR), FGFR1/2, PDGFRα/β, c‐KIT, and RET. Compound **8** was the result of further optimization and showed 95.6% FLT3 inhibition, with much lower inhibition of c‐KIT (27.2%) and KDR (9.9%). Compound **8** efficiently suppressed FLT3‐WT, FLT3‐ITD, and the drug‐resistant mutant FLT3‐D835Y, according to biochemical studies, with IC_50_ values of about 1 nM. Compound **8** demonstrated a low level of inhibition of RET (IC_50_ = 18,000 nM), FGFR1/2 (> 2,690 nM), and c‐KIT (837 nM) in comparison to gilteritinib. Over 180 kinases were found to have negligible off‐target effects by broad kinase profiling. Cell‐based research showed that FLT3‐driven AML cell lines (Molm‐14 series) had nanomolar GI_50_ values, but non‐FLT3‐driven Caki‐1 and Jurkat cells showed activity of > 1500 nM. By interacting with Cys‐694 in the active DFG‐in conformation, docking studies confirmed a type I binding mechanism. FLT3 phosphorylation inhibition in Molm‐14 cells was confirmed by Western blotting. Compound **8** showed strong binding affinity to FLT3 with a docking score of –11.283, targeting the DFG‐in active conformation [68].

To overcome resistance in AML caused by FLT3 mutations, Zhang et al. reported the synthesis and biological evaluation of a novel series of imidazo[1,2‐*a*]pyridine‐thiophene derivatives. Structural changes were made to the NEK2 inhibitor CMP3a in order to improve FLT3 inhibition and eradicate NEK2 activity. With GI_50_ values of 0.52, 0.53, and 0.57 μM, respectively, compound **9d** (Figure 7) showed strong anti‐proliferative action against FLT3‐ITD, FLT3‐D835Y, and FLT3‐F691L mutant AML cell lines. Notably, **9d** confirmed its mode of action by inducing up to 34% apoptosis in MOLM14 cells at 48 h. These substances are ATP‐competitive type‐I inhibitors, according to kinetic studies, and they can overcome activation loop‐associated resistance by binding to FLT3 in the active “DFG‐in” conformation. A homology model based on PDB: 6JOI was used for molecular docking, which revealed important interactions between **9e** and Cys694 (hinge region) and π‐stacking with Phe691 in the hydrophobic pocket [69].

**Figure 8:**
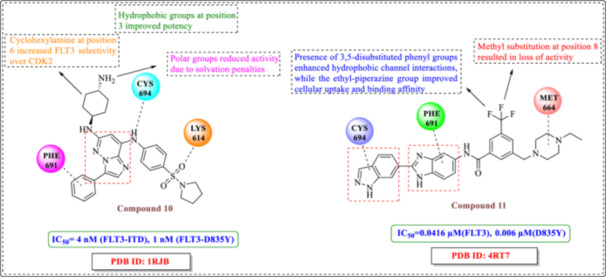
SAR of imidazole derivatives.

Petra Břehová et al. developed imidazo[1,2‐*b*]pyridazine derivatives as effective FLT3 kinase inhibitors. In FLT3‐mutant leukemic cells, these substances block FLT3 by attaching to the ATP site in its active DFG‐in conformation. This stops proliferation and triggers death by preventing the phosphorylation of downstream signaling molecules such as STAT5 and ERK. With GI_50_ values of 7–9 nM and IC_50_ values of 4 nM (FLT3‐ITD) and 1 nM (FLT3‐D835Y) in MV4‐11 and MOLM‐13 cell lines, Compound **10** (Figure 8) was found among the series in terms of inhibitory potency. An overall yield of 52% was achieved through a sequence involving Suzuki coupling and Buchwald–Hartwig amination, followed by cyclohexylamine substitution. A docking score of −11.283 kcal/mol was obtained by molecular docking against a homology model based on PDB ID: 1RJB. This indicated a selection‐favoring tight binding with important residues Cys694, Lys614, and Phe691 in the kinase pocket [70].

Im Daseul et al. synthesized a class of benzo[*d*]imidazole‐indazole compounds that are strong type II inhibitors targeting FLT3. The lead chemical **11** (Figure 8) showed impressive inhibitory efficacy with IC_50_ values of 0.0416 µM for FLT3 and 0.006 µM against the D835Y mutant. It was also able to efficiently suppress uncommon FLT3 mutations below 50 nM. After amide coupling and acid deprotection, compound **11** was synthesized, producing a high‐purity product with an overall yield of about 65%. Using the crystal structure with PDB ID 4RT7, molecular docking studies showed a strong docking score of –11.283 kcal/mol. Important interactions included hydrophobic accommodation of the ethyl‐piperazine moiety within the FLT3 binding pocket involving Met664 and Leu802, π–π stacking with Phe691 and Phe830, and hydrogen bonds with Cys694 and Asp829, among others [71].

### 5.5 Indole Derivatives as FLT3 Inhibitors

**Figure 9:**
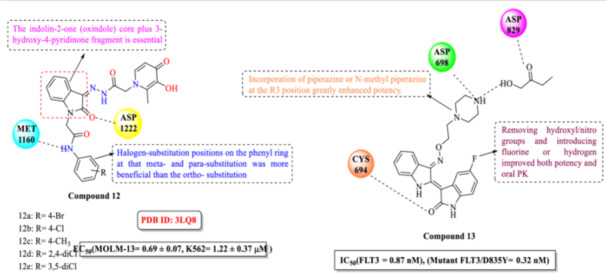
SAR of Indole derivatives.

Through a multi‐step process, the authors synthesized a series of 3‐hydrazonoindolin‐2‐one (oxindole)–3‐hydroxy‐4‐pyridinone hybrids **(12a–e).** The anticancer properties of these compounds were assessed against leukemia cell lines (MOLM‐13, K562), breast cancer (MCF‐7), and lung cancer (A549). In comparison to solid tumor lines, the library demonstrated modest antiproliferative activity, but it was particularly effective against leukemia, with **12e**(Figure 9) exhibiting sub‐micromolar activity (MOLM‐13, EC_50_ = 0.69 μM and 1.22 ± 0.37 μM against K562). Docking investigations were carried out after in silico target prediction identified kinases (including FLT3). The protocol was confirmed by re‐docking into c‐Met (PDB ID 3LQ8), which yielded an RMSD of 0.645 Å. The results of docking lead **12e** into c‐Met (3LQ8) and FLT3 (PDBs ID: 4XUF) showed favorable binding: in c‐Met, **12e** formed H‐bonds with Asp1222 and Met1160 along with extensive hydrophobic contacts (Ala1108, Met1131, Leu1140, Val1155), while in FLT3, key H‐bonds involved Cys644, Glu661, Cys694, and an additional interaction with Tyr693, along with π–π and hydrophobic packing by Phe/Tyr residues (e.g., Phe691, Phe830). Based on the combined biology and modeling data, **12e** (as well as its analogs **12a/12d**) are identified as interesting leads for additional development as FLT3/tyrosine‐kinase inhibitors related to AML [72]. When Jeong et al. synthesized derivatives of indirubin‐3′‐oxime as FLT3 inhibitors, they found that compound **13** was the most effective compound. Compound **13** demonstrated more efficacy than previously documented indirubin derivatives when tested for its inhibitory effect against FLT3 (IC_50_ = 0.87 nM) and the mutant FLT3/D835Y (IC_50_ = 0.32 nM). Additionally, it demonstrated potent antiproliferative effect against resistant MOLM14 AML cells, including FLT3‐ITD (GI_50_ = 1.85 nM), FLT3‐ITD/D835Y (GI_50_ = 1.87 nM), and FLT3‐ITD/F691L (GI_50_ = 3.27 nM), as well as MV4‐11 AML cells (GI_50_ = 1.0 nM). Crucially, at an oral dosage of 20 mg/kg, compound **13** (Figure 9) caused total tumor regression in MV4‐11 xenograft mice, shown excellent PK characteristics, and had a high oral bioavailability (42.6%) with no discernible toxicity. Compound **13** reduced the risks of hematological toxicity by preferentially inhibiting FLT3 and its mutations (> 90% inhibition) with no effect on off‐target kinases like c‐KIT (22% inhibition), according to kinase selectivity profiling at 100 nM. Its mode of action was validated by functional assays, which demonstrated suppression of downstream STAT5 and ERK phosphorylation in FLT3‐ITD AML cells. Compound **13** was docked into FLT3's ATP‐binding pocket using Discovery Studio 3.5 (CDOCKER protocol). The binding position showed several hydrophobic contacts with Leu616, Leu818, Cys828, and Tyr693, as well as two important hydrogen bonds with Cys694 (hinge area). Important hydrogen‐bonding and ionic interactions were established between the piperazine moiety and Asp698, Asn816, and Asp829 [73].

**Figure 10:**
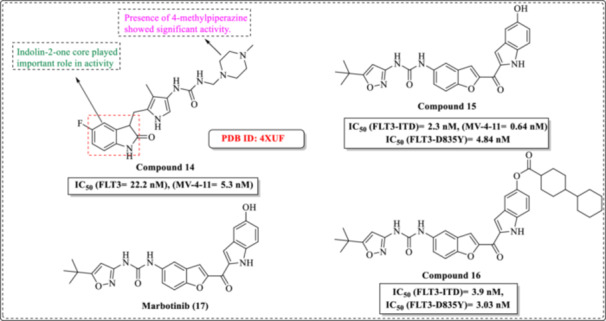
Chemical structure and SAR of Indole derivatives as FLT3 inhibitors.

Indolone derivative **14** (displayed in Figure 10) was synthesized by Jin et al. as a new FLT3 inhibitor to treat AML. Compound **14** with IC_50_ values of 8.4 nM and 5.3 nM, respectively, demonstrated outstanding efficacy against FLT3 and suppressed the growth of FLT3‐ITD positive AML cells MV‐4‐11. Compound **14** caused apoptosis in cells in a dose‐dependent manner; at doses of 10 nM, 50 nM, and 100 nM, the percentage of apoptotic MV‐4‐11 cells was, on average, 6.11%, 15.03%, and 19.02%. **14** had moderately significant antiproliferative effects against FLT3‐D835Y/D835H/D835V and substantial inhibitory efficacy against FLT3‐ITD. At dosages of 2.5, 5, and 10 mg/kg, compound **14** dose‐dependently reduced the growth of tumors in MV‐4‐11 xenograft mice. Compound 14 was docked into a FLT3 kinase with a DFG‐out conformation (PDB 4XUF). In the hinge region, the indolone scaffold established two crucial hydrogen bonds with Glu692 and Cys694. Additionally, **14** demonstrated strong complementarity inside FLT3's binding pocket. A molecular dynamics simulation lasting 100 ns was conducted using the AMBER16 software program to determine the binding stability of **14** to FLT3 [74]. Sellmer and co‐workers synthesized novel derivatives of methanones with various substituents. In compound **15**, bisindolylmethanone skeleton is embedded with ter‐butyl‐isoxazol‐urea at position 5 and contains a hydroxyl group at the terminal position. Furthermore, compound **15** was reacted with 1,4‐bipiperidine‐10‐carbonyl chloride in CH_2_Cl_2_/pyridine to yield compound **16**, which has improved water solubility. Compound **15** displayed FLT3‐ITD mutant inhibitory activity of 2.3 and 4.84 nM against FLT3‐D835Y. Moreover, compound **16** displayed FLT3‐ITD mutant inhibitory activity of 3.9 and 3.03 nM against FLT3‐D835Y. Compound **15** is a type II tyrosine kinase that stabilizes the inactive DFG‐out FLT3 conformation. The structural similarity of compounds **15** and **16** with quizartinib made these compounds more specific and active toward both FLT3 and FLT3‐ITD mutants. This data suggest that both compounds might act as novel TKI against FLT3‐ITD mutants [75]. Marbotinib is a potent FLT3 inhibitor identified through screening in FLT3‐ITD–positive leukemic cells, where it showed strong anti‐proliferative activity with low nanomolar IC_50_ values (1–5 nM in MV4‐11 cells). It effectively inhibits both FLT3‐ITD and FLT3‐TKD mutations, including resistant variants such as D835Y, showing broad activity. Mechanistically, marbotinib binds to both active and inactive conformations of FLT3, classifying it as a hybrid Type I/Type II inhibitor. In various assays, it significantly reduced cell viability and induced apoptosis, as confirmed by annexin‐V/PI staining and flow cytometry after 48 h treatment [76]. Figure 10 displays the chemical structures of various indole derivatives as FLT3 inhibitors.

### 5.6 Isoxazoles as FLT3 Inhibitors

Urea–isoxazole derivatives **18a–e** were synthesized and tested against drug resistance mutations, including FLT3‐ITD/D835Y and FLT3‐ITD/F691L as well as FLT3‐ITD mutations. Compound **18d** demonstrated broad activity with IC_50_ values of 0.072 nM (FLT3‐ITD), 3.48 nM (D835Y mutant), and 5.86 nM (F691L mutant), whereas compound **4b** was extremely active against FLT3‐ITD with an IC_50_ of 0.015 nM. By obstructing FLT3's ATP‐binding site, these compounds stop kinase activation and subsequent signaling. FLT3 was used for docking studies (PDB ID: 4RT7). Compound **18d**'s great binding affinity can be explained by the hydrogen bonds it established with Cys694, Asp829, and Glu661, as well as the π–π stacking it produced with Phe691 and Phe830 [77].

Oxazole derivatives were synthesized (**19a–d**) and tested as selective FLT3 inhibitors for possible anticancer effects. With strong inhibitory effects on FLT3‐ITD kinase (IC_50_: 0.41 nM) and MV4‐11 cell line levels (IC_50_: 0.037 μM), **19d** was found among these compounds as the most promising. The MD simulation approach was also used to propose the binding mode of **19d** with FLT3, which gave the molecule‐design justification for the extra medicinal chemistry. Compound **19d** thus serves as a novel scaffold for the subsequent identification of FLT3‐ITD inhibitors in AML treatment. Mechanistic research showed that these substances work by taking up residence in FLT3's ATP‐binding site, which prevents kinase phosphorylation and downstream signaling that is essential for leukemic cell proliferation and survival. For molecular docking investigations, the FLT3 crystal structure (PDB ID: 4XUF) was used. The most active derivative, **19d**, showed a docking score of −8.12 kcal/mol, generating π–π stacking contacts with Phe691 and stable hydrogen bonds with hinge residues Cys694 and Asp829 as well. When compared with weaker analogs that were unable to generate many stabilizing connections, its enhanced potency was explained by these strong binding interactions [78]. The authors have reported inhibitors of FLT3‐ITD and FLT3‐TKD mutants (compound **20**) and displayed excellent inhibition. But compound **20** was insoluble in water, and it also had low bioavailability. It displayed less in vivo activity as compared with quizartinib [75, 79]. To overcome this problem, the authors have incorporated some amine fragments into the aryl‐methanone‐skeleton at C‐3 or C‐4 positions of the indolyl structure. Authors have synthesized two series, bisarylmethanone and carbamate derivatives. Among all the synthesized derivatives, two compounds **21** and **22** were found to be potent FLT3 inhibitors. It has been found that the water solubility is increased by attaching an amine at an appropriate scaffold position. Compound **21** displayed an IC_50_ of 2.40 and 3.83 nM against FLT3‐ITD and FLT3‐D835Y mutants. In the second series, compound **22** displayed a potent IC_50_ of 0.81 and 0.62 nM against FLT3‐ITD and FLT3‐D835Y mutants. It has been found that prolongation of a more rigid piperazinemethyl group at 4th position leads to low activity (> 1000) [80].

**Figure 11:**
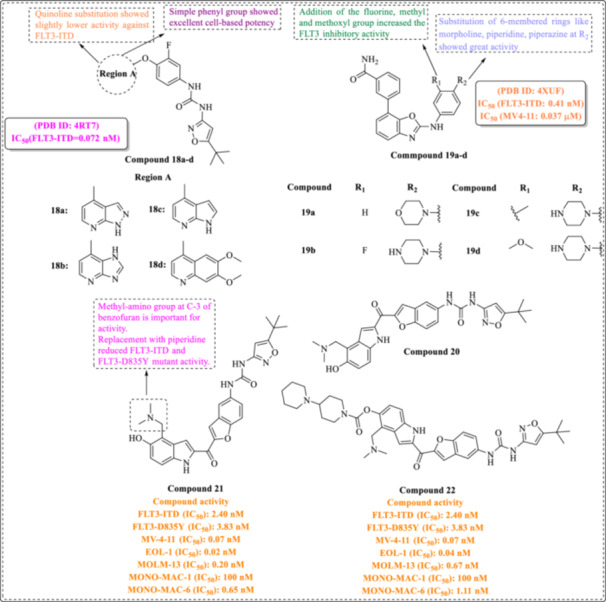
SAR of Isoxazole derivatives.

Figure 11 displays the Chemical structure and SAR of isoxazole derivatives.

### 5.7 Purine Based Derivatives

To treat AML, Tomanová et al. developed 2,7,9‐trisubstituted purin‐8‐ones as novel FLT3‐ITD inhibitors. The most active derivative of the synthesized compounds was found to be **23**. With an IC_50_ of 37 nM, it demonstrated considerable inhibitory effectiveness against FLT3 kinase and significant antiproliferative action in MV4‐11 AML cells (GI_50_ = 50 nM). It was much less effective in FLT3‐independent K562 cells (GI_50_ = 0.58 µM), indicating good selectivity. In cellular tests, **23** caused a marked G1‐phase cell‐cycle arrest by blocking downstream STAT5 and ERK1/2 signaling and inhibiting FLT3 autophosphorylation. AutoDock Vina was used to perform molecular docking on the FLT3 active DFG‐in conformation in order to better understand its mechanism. Two hydrogen bonds with Cys694 in the hinge region stabilized the type I binding mode, according to the results. Furthermore, the cyclopentyl moiety connected with Asp698 and Leu767, whereas the isopropyl group created hydrophobic contacts with Val624, Lys644, Val675, Leu767, and Phe691. All things considered, compound **23** showed strong, targeted, and well‐tolerated FLT3 inhibition, making it a viable preclinical option for AML treatment [81].

**Figure 12:**
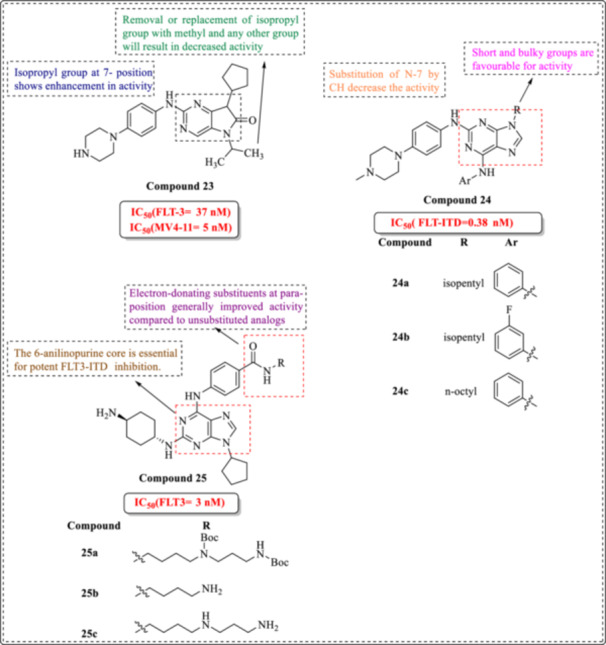
Chemical structures and SAR of purine derivatives.

Jeanluc Bertrand and colleagues synthesized 2,6,9‐Trisubstituted Purine Derivatives and assessed their anticancer efficacy against FLT3‐ITD, Bcr‐Abl, and BTK. The most effective anticancer drugs were found to be **24b** (IC_50_ = 0.38 µM for FLT‐ITD) and **24c** (IC_50_ = 0.41 µM for BTK). Two pieces, a modification at the 6‐phenylamino ring and the length and volume of the alkyl group at N‐9, were found to be strong and selective inhibitors of these three kinases by 3D‐QSAR analysis and molecular docking experiments. Before assigning scores to the new compounds, docking was confirmed by self‐docking to co‐crystal structures, FLT3 (PDB 6JQR). Compound **24b** scored −74.05 kcal/mol, and **24c** scored −61.34 kcal/mol for FLT3, with interactions in the broader hydrophobic site are representative docking/MM‐GBSA results. To improve hydrogen bonds with residue C694, **24b** demonstrated preferential hydrophobic interactions with residues V624, L616, and V675 [82]. Ristýna Vlková et al. synthesized a novel series of 2,6,9‐trisubstituted purine conjugates with diamine or polyamine chains and tested as protein kinase inhibitors. The purpose of these compounds was to block the oncogenic kinases FLT3‐ITD and PDGFRα, which are linked to both chronic eosinophilic leukemia and AML. According to biological screening, 6‐anilinopurine derivatives (**25b, 25c)** showed high cytotoxicity in MV4‐11 and EOL‐1 cells, as well as nanomolar inhibitory action against FLT3‐ITD (IC_50_ values as low as 0.002–0.010 μM) and significant PDGFRα inhibition. Molecular docking supported the biological results, showing that anilinopurines bind in the ATP pocket of FLT3 and PDGFRα with PDB ID = 6GVA. The purine core formed key hydrogen bonds with hinge residues (Cys694 in FLT3, Cys677 in PDGFRα), while the 9‐cyclopentyl group occupied the hydrophobic pocket. The 1,4‐diaminocyclohexyl moiety engaged in hydrogen bonding with Thr14 and Asp145, and polyamine chains extended toward the solvent, allowing additional electrostatic contacts with Lys/Arg residues at the entrance. Compound **25b** showed binding scores of −9.8 kcal/mol and −9.6 kcal/mol, respectively against FLT3 protein [83]. Figure 12 shows the SAR of purine derivatives.

### 5.8 Triazole‐Based FLT3 Inhibitors

**Figure 13:**
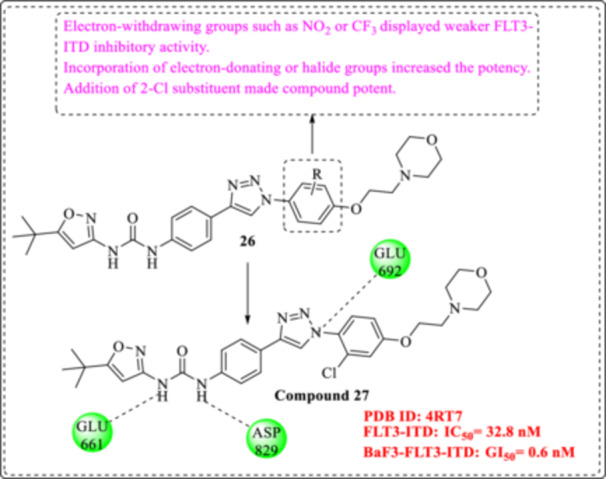
SAR of triazole derivatives.

Most of the FLT3 inhibitors displayed low or poor selectivity toward the receptor, leading to undesirable toxicity. Even, FDA‐approved drugs exhibit side effects mainly due to off‐targets. So, in the search of new candidates, Liu et al. synthesized a series of 1,4‐Diaryl‐1,2,3‐triazolo‐based urea [26] displayed in Figure 13 as novel FLT3 inhibitors. Compound **21** displayed potent FLT3‐ITD kinase inhibitory activity with an IC_50_ of 32.8 nM and BaF3‐FLT3‐ITD cell inhibitory activity with a GI_50_ of 0.6 nM. Compound **27** also showed excellent anti‐proliferative inhibitory activity against the MV4‐11 and MOLM‐13 cell lines with a GI_50_ of 3.0 and 5.9 nM. Pharmacokinetic studies displayed *T*_1/2_ of compound **27** as 6.77 h with bioavailability of 35.81%. Docking studies showed that N2 and N3 of the triazole ring formed hydrogen bond interactions with Cys694 and Glu692 amino acids. The ureido linker also formed two hydrogen bond interactions with Glu661 and Asp829 amino acids. Furthermore, the stronger hydrogen bond formed by the triazole ring in compound **27** resulted in closer contact with Phe691 than in the case of quizartinib [84].

### 5.9 Pyrimidine‐Thiazole Hybrids as FLT3 Inhibitors

**Figure 14:**
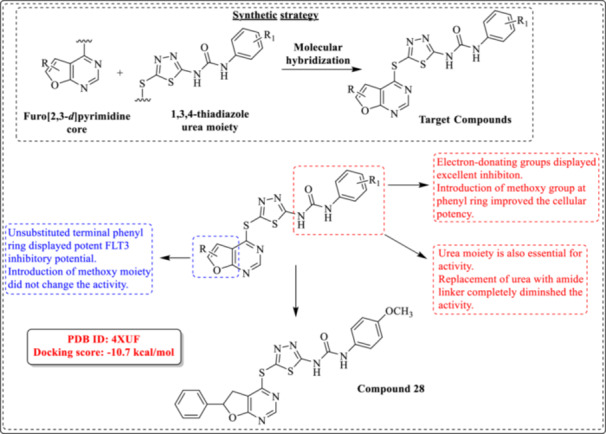
SAR of compound 28.

Moradi et al synthesized a series of furo[2,3‐*d*]pyrimidin‐4‐ylsulfanyl‐1,3,4‐thiadiazole derivatives (displayed in Figure 14) as potent FLT3‐ITD derivatives for the treatment of AML. By using a molecular hybridization approach between furo[2,3‐*d*]pyrimidine core and 1,3,4‐thiadiazole‐urea, the authors have synthesized novel derivatives. Among all the synthesized compounds, compound **28** was found to be a potent FLT3‐ITD inhibitor with an IC_50_ of 0.031 µM. Compound **28** also displayed potent antiproliferative activity against K562, MV4‐11, and MOLM‐13 cell lines with GI_50_ of 10, 0.105, and 0.063 µM. Docking studies were performed on the 4XUF protein, and the methoxyphenyl moiety of compound **1** formed hydrophobic interactions with M665 and M665. The carbonyl group of the urea moiety formed hydrogen‐bonding interactions with nitrogen of D829, and secondary amines also formed two hydrogen‐bonding interactions with E661. The thiadiazole ring formed pi–pi interactions with F691. Compound **28** also induced cell‐cycle arrest in the G1 phase of the cell cycle. This study showed that substituted furo[2,3‐*d*]pyrimidines compounds could be used as FLT3 inhibitors as targeted therapy in AML [85].

### 5.10 Pyrazole as FLT3 Inhibitors

4‐[(6‐Phenoxypyrimidin‐4‐yl)‐amino]‐*N*‐(4‐(piperazin‐1‐yl)phenyl)−1*H*‐pyrazole‐carboxamide was created and tested for its ability to inhibit cancer in response to FLT3‐ITD and FLT3‐TKD. Compound **23** demonstrated superior anti‐proliferative properties against BaF3 cells using (FLT3‐ITD, D835V) (IC_50_ = 9 nM), (FLT3‐ITD, F691L; IC_50_ = 95 nM), (FLT3‐ITD, N676D; IC_50_ = 54 nM), and FLT3 (D835V; IC_50_ = 43 nM). Compound **29** established two hydrogen bonds with Glu692 and Cys694 in the hinge region of FLT3 through molecular docking using the FLT3 homology model (DFG‐in) and crystal structure (PDB ID: 6JQR). With the help of Leu646, Met665, Leu832, Phe830, and Asp829, the phenoxy group of **29** expanded to the rear pocket and created advantageous hydrophobic contacts [86].

Derivatives of biphenyl‐substituted pyrazolyl‐urea were created and tested for anticancer properties as new FLT3 inhibitors for AML. Compound **30d** demonstrated strong activity against recombinant FLT3 among all synthesized derivatives, with IC_50_ values of 280 nM in MV4.11 cells and 18 nM in MOLM‐14 cells. Its inactivity against control HL60 cells confirmed its selectivity. In testing for metabolic stability, **30d** demonstrated intermediate stability in human microsomes; kinase selectivity profiling indicated activity primarily against FLT3 and c‐KIT; and apoptosis experiments showed that it strongly promoted cell death by decreasing the phosphorylation of FLT3 and STAT5. PDB ID 4RT7 was used for docking investigations (FLT3 bound with quizartinib). In a position that was very similar to that of quizartinib, compound **30d** docked and developed important contacts. Its urea group engaged the Lys644–Glu661 salt bridge, its acetyl group made a strong hydrogen bond with Cys694 of the hinge region, and its core aromatic ring π‐stacked with Phe691 and Phe830. Docking and molecular dynamics confirmed steady binding and numerous hydrogen bonds, which explained its high affinity [87].

**Figure 15:**
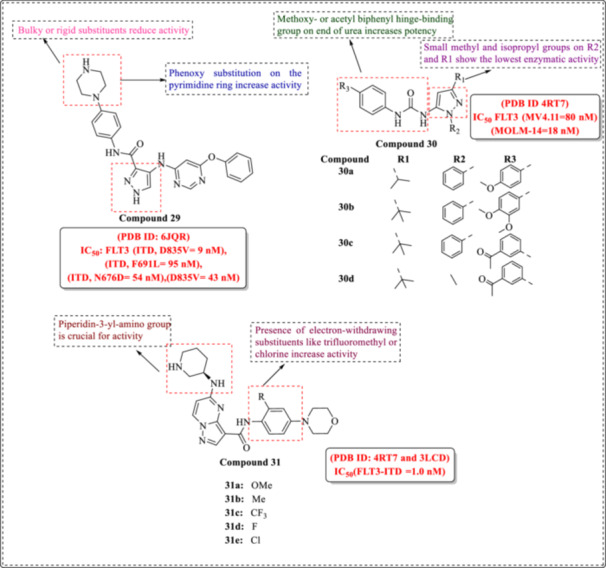
Chemical structure and SAR of pyrazole derivatives.

Synthesized pyrazolo[1,5‐a]pyrimidines were tested as potent FLT3‐ITD inhibitors in AML(AML). Among all of them, Compound **31c** performed exceptionally well. At very low concentrations IC_50_ values of roughly 1.0 nM, compound **31c** inhibited FLT3‐ITD activity. They also inhibited the development of AML cells (MV‐4‐11 and MOLM‐13) with IC_50_ values of 1.3 and 0.4 nM, outperforming quizartinib. Protein Data Bank FLT3 protein models (PDB ID: 4RT7 and 3LCD) were used for docking investigations. According to docking data, compound **31c** is located in FLT3's ATP‐binding pocket. For stable binding, it establishes crucial hydrogen bonds with the hinge area residues Cys694 and Asp829, which are essential. Furthermore, the phenyl ring's trifluoromethyl group enhances affinity by interacting with the solvent‐exposed area. The binding is further stabilized by hydrophobic interactions with residues Phe691, Leu818, and Val624 [88]. Figure 15 displays the chemical structure and SAR of pyrazole derivatives as FLT3 inhibitors.

### 5.11 In Silico Approaches Used to Identify FLT3 Inhibitors

In silico approaches have been extensively applied to accelerate the identification and optimization of FLT3 inhibitors, especially given the high cost and time associated with experimental screening. These computational methods enable the exploration of large chemical libraries, prediction of binding affinities, and detailed analysis of molecular interactions with the ATP‐binding site of FLT3. Techniques such as molecular docking, pharmacophore modeling, and virtual screening are commonly employed to identify potential leads, while molecular dynamics simulations and free energy calculations provide deeper insights into the stability and binding mechanisms of inhibitor–FLT3 complexes. Table 3 displays the various in silico approaches used for the identification of potent FLT3 inhibitors.

**Table 3:** Summary of in silico approaches used for identification of potent FLT3 inhibitors.

S. No.	In silico approach used	Database/No. of compounds screened	Potent compound	Docking score (−kcal/mol)	RMSD value (Å)	MM‐GBSA results (−kcal/mol)	Reference
1	Pharmacophore modeling, Molecular docking, MD simulation, MM‐GBSA	COCONUT (4,00,000)	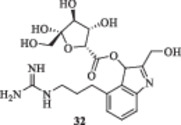	−16.041	3.2	−73.75	[89]
2	Pharmacophore modeling, Virtual Screening, Molecular docking, MD simulation	ZINC (23 million)	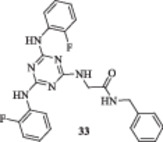	−11.5	3.6	—	[90]
3	Molecular docking, MD Simulations, MM‐GBSA	Literature (40 compounds)	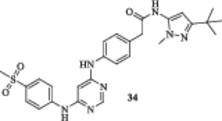	−11.31	3.7	−62.80	[91]
4	Molecular docking, MD Simulations, MM‐GBSA, CoMFA and CoMSIA Studies	Literature (47 compounds)	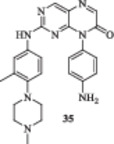	−6.93	2.3	−53.90	[92]
5	Molecular docking	ChemDiv (1.5 million)	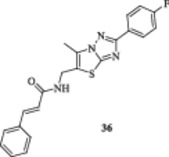	−10.84	—	—	[93]

### 5.12 PROTACs as Potent FLT3 Inhibitors

**Figure 16:**
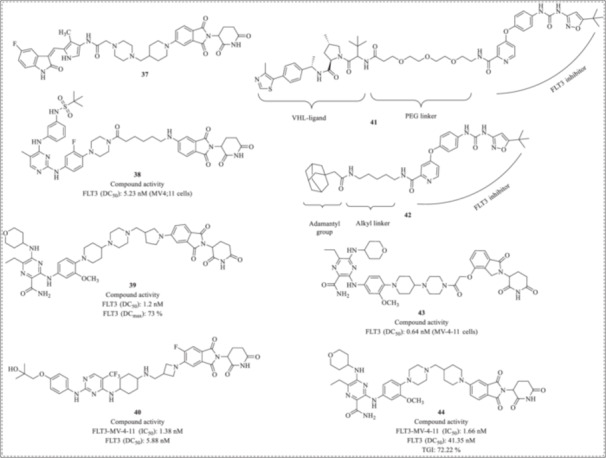
Recently synthesized PROTACs as FLT3 inhibitors.

Wang et al. synthesized an orally bioavailable FLT3 PROTAC degrader, **37** for the treatment of AML. PROTAC is one of the important approaches to eliminate the resistance of FLT3 inhibitors. The synthesized PTOTAC displayed biological activity on AML xenograft models, and also this compound is found to eliminate the CD45^+^CD33^+^ cells in murine [94]. Xiang and co‐workers synthesized a new FLT3 inhibitor, and compound **38** was found to be a potent FLT3 inhibitor with DC_50_ of 5.23 nM. It also inhibited the MV4;11 cell proliferation and displayed potent antitumor activity against FLT3 mutant Ba/F3 cells [95]. Another FLT3 degrader was synthesized by Yang et al., and compound **39** was found to be a potent FLT3 PROTAC degrader with a DC_50_ value of 1.2 nM and maximum degradation of 73%. Compound **39** also induces degradation of GSPT1 and IKZF1/3. Cotreatment of compound **39** with MG132 displayed a proteasome‐dependent degradation mechanism. Compound **39** also displayed good anticancer activity on FLT3‐ITD mutant cell lines such as MV4‐11 and MOLM‐13 with IC_50_ of 1.67 and 3.16 nM with reference to gilteritinib. It also displayed favorable PK characteristics with a half‐life of 21.58 h [96]. Lian and co‐workers also synthesized dual FLT3 and CHK1 (PROTACs) for the treatment of AML, and compound **40** displayed potent FLT3 degradation (DC_50_) of 5.88 nM. Antiproliferative activity was also carried out on MV‐4‐11 cell lines, and compound **40** displayed an IC_50_ of 1.38 nM [97]. Halilovic and co‐workers evaluated the FLT3 degradation potential of two compounds **41** and **42**. These compounds are structurally different in VHL ligand and linkers. Compound **41** is composed of a VHL ligand and PEG linker, whereas compound **42** contains adamantyl as ligand and an alkyl linker. It has been found that PROTAC **41** and FLT3 hydrophobic tagging compound **42** decreased the endoplasmic reticulum‐associated oncogenic FLT3‐ITD. Both of the compounds also induce apoptosis in leukemia cell lines [98]. Liu and co‐workers synthesized a potent and selective FLT3 PROTAC degrader for the treatment of AML. Compound **43** displayed excellent FLT3 degradation in MV4‐11 cells (with FLT3‐ITD mutations) with a DC_50_ value of 0.64 nM and *D*_max_ of 94.8%. It also induced cell‐cycle arrest in the G0/G1 phase with the proportion of 79.8% and 82.1% in MV4‐11 cells [99]. Ye and co‐workers synthesized potent gilteritinib‐based FLT3‐PROTAC degraders for the treatment of AML. They synthesized a series of degraders by tethering gilteritinib to thalidomide via different linkers. Introduction of an aliphatic heterocyclic ring as a linker displayed good antiproliferative activity. Among all the synthesized compounds, compound **44** was found to be a potent antiproliferative agent against the MV‐4‐11 cell line, displaying an IC_50_ of 1.66 nM. Further, FLT3‐ITD degradation study was carried out, and compound **44** showed DC50 values of 41.35 and 89.18 nM against MV‐4‐11 and MOLM‐13 cells. In vivo results displayed TGI of 53.54% at 5 mg/kg dose. But, when this dose was increased to 10 mg/kg, TGI reached 77.22%. Finally, antiproliferative effect was checked in different FLT3‐resistant mutant Ba/F3 cells, and compound **44** displayed IC_50_ in the range of 5.45 to 102.44 nM [100]. Chemical structures of various PROTACs as FLT3 are displayed in Figure 16.

## 6 Prominent Pharmacophoric Scaffolds as Potent FLT3 Inhibitors

**Figure 17:**
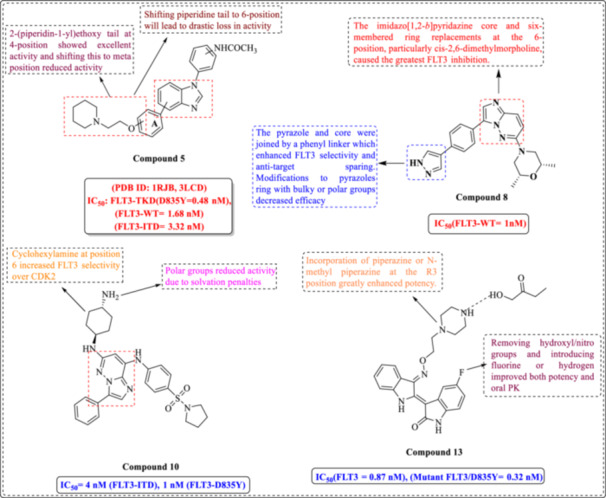
Top prominent pharmacophore scaffolds and their derivatives as potent FLT3 inhibitors.

The identification of potent FLT3 inhibitors has been the focus of extensive medicinal chemistry research. Among the various scaffolds evaluated, several have shown remarkable inhibitory activity and have been recognized as prominent pharmacophoric frameworks. The benzimidazole scaffold present in compound **5** has exhibited strong FLT3 inhibition due to its ability to establish key interactions within the ATP‐binding site of the kinase. Similarly, imidazole‐based compounds such as **8** and **10** have also exhibited significant inhibitory potential. The imidazole ring is known for its favorable physicochemical properties and its ability to form hydrogen bonds and π–π stacking interactions, which leads to its high binding affinity. Indole is another important scaffold present in compound **13**. It is a widely recognized scaffold in drug design because of its planar aromatic structure and potential to engage in various non‐covalent interactions with biological targets. Its incorporation in compound **13** has been associated with strong FLT3 inhibition. Together, these scaffolds benzimidazole, imidazole, and indole represent some of the most promising pharmacophoric cores for the development of FLT3 inhibitors. Their structural features and interaction profiles may serve as a foundation for the rational design of novel, more effective therapeutic agents targeting FLT3‐driven malignancies. Figure 17 displays the prominent pharmacophoric scaffolds as potent FLT3 inhibitors.

## 7 Conclusion and Future Perspective

FLT3 remains a critical therapeutic target in AML due to its central role in hematopoietic signaling and its frequent mutation‐driven activation in malignancy. The past 5 years have witnessed remarkable progress in the development of FLT3 inhibitors, marked by the approval of second‐generation agents such as quizartinib and gilteritinib, and the emergence of novel scaffolds exhibiting improved selectivity and potency against resistant mutations. Advancements in medicinal chemistry have expanded the chemical space with promising scaffolds such as benzimidazole, imidazole enabling more effective targeting of the ATP‐binding pocket and overcoming resistance mechanisms. This review has explored updated insights into FLT3 signaling biology, clinical trial and medicinal chemistry advancements to provide a comprehensive understanding of the current therapeutic landscape. It also highlights key compounds demonstrating potent FLT3 inhibition and explores structural features contributing to their efficacy. Despite these advances, resistance to FLT3 inhibitors remains a formidable challenge, often driven by secondary mutations and activation of compensatory signaling pathways. Future efforts must therefore prioritize a deeper mechanistic understanding of resistance, alongside the design of next‐generation inhibitors capable of circumventing these hurdles. Rational combination therapies targeting parallel oncogenic pathways may also enhance therapeutic durability and prevent relapse. Looking ahead, structure‐guided drug design, integrated with cutting‐edge computational modeling and high‐throughput screening, will be vital in refining inhibitor specificity and minimizing off‐target effects. Furthermore, expanding the application of FLT3 inhibitors beyond AML to other FLT3‐altered malignancies offers a promising avenue to broaden their clinical impact. In conclusion, the evolving landscape of FLT3‐targeted therapies holds significant promise for improving patient outcomes. Continued interdisciplinary collaboration across medicinal chemistry, molecular biology, and clinical oncology will be essential to translate these insights into personalized, more effective anticancer strategies.

## Funding

The authors have nothing to report.

## Conflicts of Interest

The authors declare no conflicts of interest.

## Data Availability

Data sharing is not applicable to this article as no datasets were generated or analyzed during the current study.
